# Utility of Teacher-Report Assessments of Autistic Severity in Japanese School Children

**DOI:** 10.1155/2013/373240

**Published:** 2013-12-11

**Authors:** Yoko Kamio, Aiko Moriwaki, Naoko Inada

**Affiliations:** Department of Child and Adolescent Mental Health, National Institute of Mental Health, National Center of Neurology and Psychiatry, 4-1-1 Ogawa-Higashi, Kodaira, Tokyo 187-8553, Japan

## Abstract

Recent studies suggest that many children with milder autism spectrum disorder (ASD) are undiagnosed, untreated, and being educated in mainstream classes without support and that school teachers might be the best persons to identify a child's social deviance. At present, only a few screening measures using teacher ratings of ASD have been validated. The aim of this study was to examine the utility of teacher ratings on the Social Responsiveness Scale (SRS), a quantitative measure of ASD. We recruited 130 participants aged 4 to 17 years from local schools or local pediatric outpatient clinics specializing in neurodevelopmental disorders that included 70 children with ASD. We found that the teacher-report SRS can be reliably and validly applied to children as a screening tool or for other research purposes, and it also has cross-cultural comparability. Although parent-teacher agreement was satisfactory overall, a discrepancy existed for children with ASD, especially for girls with ASD. To improve sensitivity in children at higher risk, especially girls, we cannot overstate the importance of using standardized norms specific to gender, informant, and culture.

## 1. Introduction

The current professional consensus is that early diagnosis and subsequent early treatment of autism spectrum disorder (ASD) can facilitate development and learning [1, 2], reduce the need for treatment later in life [3, 4], and improve longterm prognosis in adulthood [5, 6]. However, not all families with children with ASD necessarily get timely access to treatment and other support. Delayed identification and diagnosis of ASD have been associated with subtypes of ASD [7–10], cognitive level [10, 11], gender [11, 12], and demographic factors such as low socioeconomic status [8, 10]. Diagnosis of ASD tends to be delayed in children having both milder autistic symptoms and above-average general cognitive ability, especially in girls. For example, reported age at first diagnosis of Asperger, syndrome ranges from 7 to 11 years [9, 10, 12, 13]. In a Japanese nationwide survey of adults with high-functioning ASD, the median age at first diagnosis was 10.3 years [6].

Recent epidemiological studies [14, 15] have revealed that most mainstreamed children with ASD were undiagnosed and untreated. Although most of these children might have had few diagnosable symptoms during preschool to draw the attention of primary health professionals, school teachers should be the best persons to identify any overt social deviance [16, 17].

At present, many quantitative behavioral measures of ASD have been created and validated in both primary care and clinical settings. However, these measures were largely validated for use by parents, not teachers, except in the case of the Autism Spectrum Screening Questionnaire (ASSQ), the Social Communication Questionnaire (SCQ), and the Social Responsiveness Scale (SRS). The ASSQ is a 27-item questionnaire that was originally developed as a first-stage population screening instrument in a prevalence study of Asperger, syndrome in mainstream schools with teachers as target raters [18], and it has been validated as a general population screen [19, 20]. The reliability and validity of both parent and teacher ASSQ ratings in a clinical setting have also been reported, although parent-teacher agreement was low to moderate for children with high-functioning ASD [21]. The SCQ [22] is a 40-item screening instrument that has been investigated mainly as a parent-report screen. In one study of children with ASD and their siblings [17], the teacher-report SCQ-Current version was moderately correlated with the parent-report SCQ-Lifetime version, whereas it was strongly correlated with the teacher-report SRS. The SRS was developed as a quantitative measure of autistic traits in children [23], and the parent-report SRS has been extensively validated for the general child population [24–27] as well as for clinical samples [24, 28–32] not only in the USA but also in Europe, South America, and Asia. On the other hand, the literature on the utility of the SRS as a screening tool assessed by teachers is still limited [17, 31, 33]. Constantino et al. [34] demonstrated that the teacher-report SRS exhibited strong correlations with the parent-report SRS (*r* = 0.72), and the combined use of both parent and teacher reports resulted in extremely high sensitivity and specificity for a diagnosis of ASD in 271 children with ASD and 171 children without ASD, including 52 child psychiatric patients and 119 unaffected siblings. Schandling et al. [17] examined the utility of parent- and teacher-report SCQ and SRS in 1,663 children with ASD and 1,712 unaffected siblings from 1,655 families and showed that the screening properties of the teacher-report SRS were superior to those of the teacher-report SCQ-Current. In their study, the teacher-report SRS was more congruent with clinician-observed behaviors than with parent-reported behaviors and raised the possibility that behaviors exhibited by the children with ASD are contextually related and might be more congruent across classroom and clinical settings [17]. Fombonne et al. [31] examined the psychometric properties of the SRS-Spanish version in a Mexican sample consisting of 140 children with ASD and 319 community controls and found that the teacher-report SRS was an excellent screening tool similar to the parent-report SRS. In addition, they noted that the parent-teacher correlation of the SRS was much higher in the ASD sample compared with the control group.

Although some evidence exists on the SRS as a screening tool assessed by teachers, its utility has not been examined in an Asian population. Further, the reason for the discrepancy between parent and teacher reports on this scale is unclear.

Thus, the main aim of this study was to examine the utility of the SRS-Japanese version as a teacher-report screening tool for ASD. To this end, we examined test-retest reliability and discriminant/convergent validity of the teacher-report SRS, parent-teacher correlations or discrepancies on the SRS, and screening cutoffs in Japanese children aged 4 to 17 years.

## 2. Materials and Methods

### 2.1. Participants

This study involved 130 children consisting of 70 children with ASD (51 boys, mean age 8.6 [3.7], range 4–17 years) and 60 children without ASD (39 boys, mean age 8.0 [2.5], range 5–15 years; 24 with any neuropsychiatric diagnosis other than ASD; and 36 typically developing [TD] children). Seventy-eight children (23 with ASD, 19 with any neuropsychiatric diagnosis, and 36 with TD) currently participated in our ongoing community-based longitudinal study of child mental health at the National Center of Neurology and Psychiatry (NCNP), Japan. All research participants were attending mainstream classes at local schools. We also recruited 20 children from a local special school for children with learning disabilities (15 with mental retardation [MR] and ASD, 5 with MR only). In addition, we recruited 32 patients diagnosed with ASD from three local pediatric outpatient clinics specializing in neurodevelopmental disorders.

The gender ratio did not significantly differ between children with ASD and those without ASD (*χ* ^2^ = 0.94, ns). Mean age did not significantly differ between groups (*t* = 1.16, ns).

### 2.2. Measures

#### 2.2.1. The Social Responsiveness Scale

The SRS is a 65-item questionnaire of autistic traits for use with 4–18-year-olds that can be completed in 15 minutes by parents or teachers who have observed the child over time in naturalistic social settings [23]. The SRS was developed to assess autistic symptoms or quantitative traits and has subsequently undergone extensive validation in general and clinical child populations in the USA and other countries. The 65 SRS items can be categorized into five subscales (social awareness, social cognition, social communication, social motivation, and autistic mannerisms). Each item is scored on a 4-point scale, and total score ranges from 0 to 195, with higher scores indicating higher degrees of social impairment. We used the teacher version in the present study and also the parent version as a subsample. The Japanese version of the parent SRS exhibited a skewed normal distribution in the general population with a single-factor structure, had no relation to IQ within the normal intellectual range [27], and demonstrated satisfactory discriminant and convergent validity [27, 35]. Both the parent- and teacher-report SRS were standardized on boys and girls separately [36].

#### 2.2.2. The Autism Diagnostic Interview-Revised (ADI-R)

The Autism Diagnostic Interview-Revised (ADI-R) [37] is a parent-report interview and a research standard for establishing a diagnosis of autism. The algorithm generates scores in each of three domains: reciprocal social interaction; communication; and restricted, repetitive, and stereotyped patterns of behavior. We used total scores of three domains of the Japanese version of the ADI-R [38] for the analysis in this study.

#### 2.2.3. The Autism Diagnostic Observation Schedule (ADOS)

The ADOS [39] is a semistructured behavioral assessment of social interaction, communication, and stereotyped behaviors. The algorithm generates scores in each of the three domains. We used total scores of the social and communication domains of the Japanese version of the ADOS [40] for the analysis in this study.

### 2.3. Procedure

The study protocol was approved by the Ethics Committee of the NCNP. A written informed consent was obtained from the parents of each child participant, and the study was conducted from 2010 to 2012.

First, parents were informed about the study by a letter from the investigators, which was distributed by the investigators themselves, a principal teacher, child psychiatrist, or pediatrician. Second, after providing the written consent, parents asked classroom teachers to complete the SRS on their children. Among all returned questionnaires, we excluded 16 teacher reports (11.0%) that had one or more missing answers, leaving 130 teacher reports on 130 children. Among these, we obtained 109 parent reports on 109 children (57 with ASD, 52 without ASD [19 clinical, 33 TD]).

Our research team conducted diagnostic interviews at the NCNP for 78 children, at the special school for 20 children, and at clinics for 32 children.

ASD diagnoses were confirmed according to DSM-IV-TR criteria based on all available clinical information by our research team that included experienced child psychiatrists and licensed clinical psychologists. To corroborate each ASD diagnosis, we evaluated the severity of autistic symptoms using either the Japanese versions of the Autism Diagnostic Interview-Revised (ADI-R) [38], the Autism Diagnostic Observation Schedule (ADOS) [40], the Diagnostic Interview for Social and Communication Disorders [41], or other semistructured interviews developed and validated in Japan [42]. Among 70 children with ASD, 55 were subcategorized with 100% diagnostic agreement based on available information among our research team: 24 with autistic disorder, 10 with Asperger's disorder, and 21 with pervasive developmental disorder, not otherwise specified. For 15 children, we reached complete agreement on a diagnosis of ASD, although we could not reach agreement on the subcategory.

The non-ASD diagnoses of 24 children were attention deficit hyperactivity disorder (ADHD), oppositional defiant disorder, specific phobia, social phobia, obsessive-compulsive disorder, enuresis, tic disorder, or mental retardation. These diagnoses were confirmed by diagnostic interviews with children and their parents using the Kiddie Schedule for Affective Disorder and Schizophrenia Present and Lifetime (K-SADS-PL), Japanese version. By parent interview, we confirmed the typical development of 36 children as having no history of neurological or psychiatric disorders.

We judged intellectual level based on cognitive testing (i.e., various versions of the Wechsler Intelligence Scale or other measures) for 115 children and educational/administrative records for 15 children. Intellectual level ranged from normal intelligence to severe MR (normal to borderline 105, mild MR 8, moderate MR 6, severe MR 4, and unknown MR 7). The proportion of children with normal intelligence did not significantly differ between children with ASD (53/70) and those without ASD (52/60) (*χ* ^2^ = 2.5, ns).

### 2.4. Data Analysis

To address discriminant validity, we compared mean total and mean subscale SRS scores by gender between children with ASD (*n* = 70) and those without ASD (*n* = 60). To examine test-retest reliability, we calculated the intraclass correlation coefficient (ICC) for a subsample (*n* = 23). To examine convergent validity, we computed Pearson's correlation coefficients between the SRS and ADI-R, ADOS, or full scale IQ scores on three subsamples (*n* = 49, 56, 115). To examine the teacher-parent discrepancy, we calculated ICC and compared mean total and mean subscale SRS scores by group (ASD versus non-ASD) and by gender using a paired *t*-test on a subsample (*n* = 109) that included both teacher and parent ratings. Finally, we conducted a receiver operating characteristics (ROC) analysis to compare the area under the curve (AUC) for the parent- and teacher-report SRS for a subsample (*n* = 109), and determined the cutoff scores that maximized sensitivity and specificity for the teacher-report SRS for the total sample.

All analysis was performed using SPSS 18.0J for Windows.

## 3. Results

### 3.1. Discriminant Validity

Table 1 presents the mean raw teacher-report SRS scores for the total sample (*N* = 130; ASD 70, non-ASD 60 [non-ASD diagnosis 24, TD 36]) by gender. Total scores and the five subscale scores were significantly higher in children with ASD than in those without ASD for both genders, except for social awareness and social motivation subscales in girls, where the mean subscale scores did not significantly differ between girls with ASD and those without ASD.

### 3.2. Test-Retest Reliability

Among 130 children, 23 (ASD 1, non-ASD diagnosis 3, TD 19) were assessed by their classroom teachers on two occasions with a mean interval of 40.0 days (12–131 days). Test-retest reliability was shown to be excellent for the total score (time 1: mean SRS 63.2 [22–103]; time 2: mean SRS 61.4 [24–119]; ICC = 0.87; *P* < 0.001).

### 3.3. Convergent Validity

SRS total score was significantly positively correlated with ADI-R total score (*r* = 0.30, *P* < 0.05) in a subsample with available data from both the SRS and ADI-R (*n* = 56, ASD 21, non-ASD diagnosis 14, TD 21; 36 boys) and also significantly correlated with ADOS total score (*r* = 0.30, *P* < 0.05) in a subsample with available data from both the SRS and ADOS (*n* = 49, ASD 20, non-ASD diagnosis 16, TD 13; 35 boys). In 115 children with available IQ data, the SRS score did not significantly correlate with IQ (*r* = −0.14) for 97 children with IQs ≥ 70 (ASD 46, non-ASD diagnosis 16, TD 35; 66 boys), whereas it significantly correlated with IQ in 18 children with IQs < 70 (ASD 11, non-ASD diagnosis 7) (*r* = −0.58, *P* < 0.05).

### 3.4. Parent-Teacher Correlation and Discrepancy

Among 130 total participants, 109 participants (ASD 57, non-ASD 52, including non-ASD diagnosis 19 and TD 33) were rated by both their teachers and parents at almost the same time (Table 2). For this subsample (73 boys, mean age 10.9 [2.8], range 7–14 years), ICCs showed moderate to large agreement between teachers and parents for all 109 children (73 boys and 36 girls; ICCs = 0.48, 0.50, and 0.40, resp.; Table 3). Among five subscales, ICCs ranged from moderate to large (ICCs = 0.29–0.53, *P* values <0.05), except for the social awareness subscale in girls (ICC = 0.08, ns).

Table 4 shows that children with ASD of either gender were rated significantly higher by parents than by teachers on the total scores. Among five subscales, significant differences in ratings between parents and teachers were found only for autistic mannerisms in boys with ASD, whereas subscale ratings on social cognition, social communication, and autistic mannerisms were significantly different in girls with ASD. On the other hand, children without ASD of either gender were rated similarly by parents and teachers on the total scale and on all subscales.

For children with ASD, we found a significant gender difference in teacher ratings on the SRS only on the social awareness subscale, where teachers rated girls significantly lower than boys (*t* = 2.10, *P* < 0.05). By contrast, we found no significant gender differences in parent ratings for this sample. On the other hand, for children without ASD, we observed no significant gender differences in both parent and teacher ratings (Table 4). That is, the gender difference was strongest in teacher reports on social awareness in the ASD group. Thus, teachers tended to rate boys and girls with ASD lower compared to parents, and teachers tended to rate girls with ASD lower compared to boys with ASD.

### 3.5. ASD Cutoff Scores

**Figure 1:**
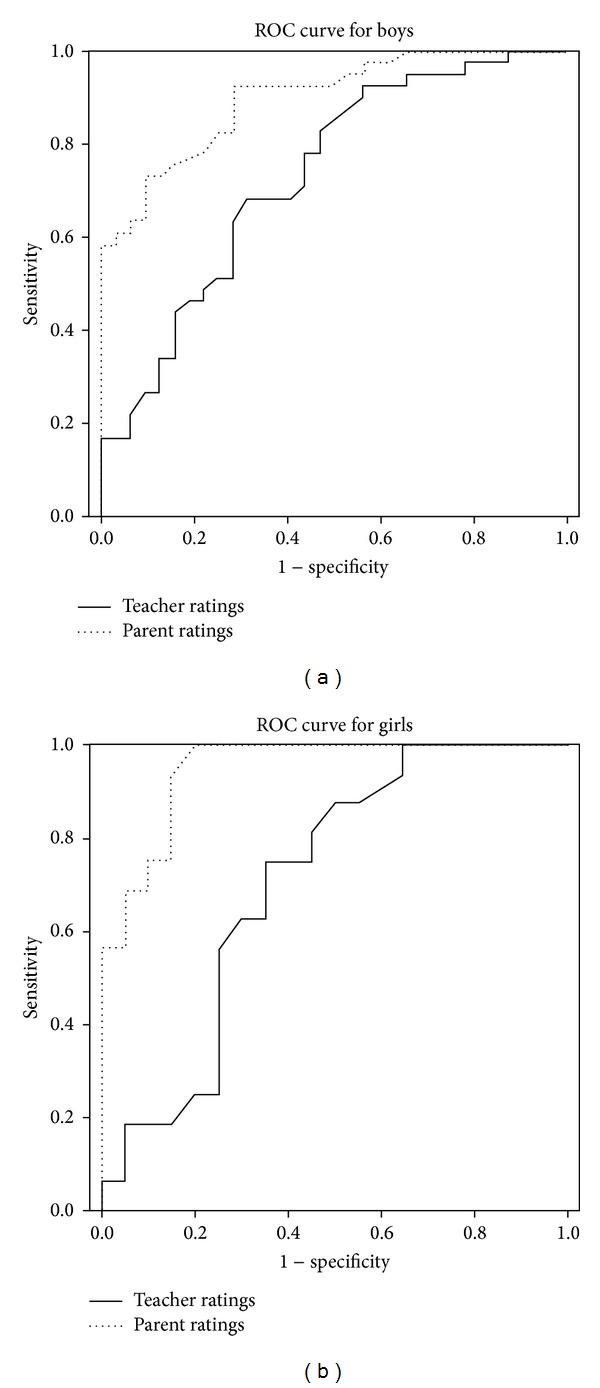
(a) Receiver operating characteristics (ROC) curve demonstrating sensitivity and specificity of both teacher and parent ratings for boys (*n* = 73). (b) Receiver operating characteristics (ROC) curve demonstrating sensitivity and specificity of both teacher and parent ratings for girls (*n* = 36).

ROC analyses of 109 children who were rated by both parents and teachers informed the AUC for each parent and teacher report on the SRS; among this sample, the teacher-report SRS accurately classified 73.2% of boys (*P* < 0.005) and 70.8% of girls (*P* < 0.05), whereas the parent-report SRS accurately classified 90.0% of boys (*P* < 0.005) and 94.8% of girls (*P* < 0.005) (Figures 1(a) and 1(b)). Therefore, the parent-report SRS appears to be more accurate than the teacher-report SRS as a screening tool. For the total sample, Youden's index was computed to determine the cutoff points that maximized the sum of sensitivity and specificity of the teacher-report SRS, 58.0 for boys (sensitivity 0.725, specificity 0.667, false-negative rate 0.275, false-positive rate 0.333, and positive likelihood ratio 2.177) and 43.0 for girls (sensitivity 0.789, specificity 0.667, false-negative rate 0.211, false-positive rate 0.333, and positive likelihood ratio 2.369). These optimal cutoff scores were found to correspond to a *T*-score of 60 for each boy and girl according to *T*-score norms that were created for the Japanese standardization sample [36]. Because no natural cutoff was found that differentiated children diagnosed with ASD from those without ASD in the Japanese general and clinical samples for the parent-report SRS [27], these cutoff scores of teacher-report SRS would identify many subthreshold conditions and at the same time miss many true-positive children. Compared to the previously reported optimal cutoff scores on the parent-report SRS (boys, sensitivity 0.91, specificity 0.48, and positive likelihood ratio 1.75; girls, sensitivity 0.89, specificity 0.41, and positive likelihood ratio 1.51) [27], the optimal cutoff scores on the teacher-report SRS would seem to result in a higher false-negative rate (boy, 0.28 versus 0.09, girl, 0.21 versus 0.11, teacher, and parent, resp.) and a lower false-positive rate (boy, 0.33 versus 0.52, girl, 0.33 versus 0.59, teacher, and parent, resp.). As addressed by Constantino et al. [34], when both parent and teacher rate a child as having a *T*-score of ≥60, the positive likelihood ratio would improve up to 3.730 in our sample, which exceeds the teacher-report SRS alone or the parent-report SRS alone.

## 4. Discussion

The main aim of this study was to examine the utility of the teacher-report SRS as an ASD screening tool for Japanese children. In this study, the teacher-report SRS demonstrated excellent test-retest reliability and satisfactory discriminant and convergent validity for measuring autistic severity in children aged 4 to 17 years. Overall, there were moderate to large parent-teacher correlations on the total and subscale ratings. Thus, our findings showed that the teacher ratings on the Japanese version of the SRS can be reliably and validly applied to Japanese children at school or in clinical settings as a screening tool of ASD clinical settings.

Our results suggest overall good agreement on SRS measurements in terms of severity of autistic symptoms between teachers and parents; the correlations fall within the range reported in previous studies for the SRS (0.24–0.82) [17, 29–31, 33, 34, 43]. Our result is satisfactory compared to other psychiatric domains [43]. However, it is difficult to compare ours with the correlations reported by previous studies because of differences in sample size (26–3375), the proportion of children with ASD included in the total sample (0–69.5%), and how control children were sampled (siblings from families who registered participation in autism research, community schools, and clinical non-ASD psychiatric patients); there appears to be no systematic tendency explaining the wide variation. For example, in Fombonne et al. [31], parent-teacher correlations for total SRS were stronger in children with ASD than in control children, but the opposite was found in Kanne et al. [43]. Based on data from Japan, the correlation for the non-ASD sample (Pearson's *r* = 0.78) [35] decreased to an ICC of 0.48 when calculated for the sample that included children with ASD (52.3%) in this study. Further studies are needed to answer this issue.

Despite overall good agreement, teachers tended to rate both boys and girls with ASD lower than did parents, although the teacher-parent discrepancy was not pronounced in children without ASD. Such discrepancy relating to the type of children (ASD versus non-ASD) was consistently found in previous studies [17, 34, 43, 44] except in a study based on a Mexican sample [31]. In the present study, teacher-parent discrepancy was pronounced, especially for girls with ASD (teacher 62.3 versus parent 85.1); parent ratings were significantly higher than teacher ratings not only on the total score but also on 3 (social cognition, social communication, and autistic mannerisms) of 5 subscales. One possible interpretation could be an effect of situational context as suggested by Szatmari et al. [45] and Posserud et al. [19]. How children with ASD behave can change depending on the situation, such as the degree of structurization, and it is likely that autistic behaviors of higher-functioning children with ASD are observed less often at school than at home if the school environment meets a child's needs. Shanding et al. [17] raised the possibility that teachers and clinicians similarly observe and report behaviors exhibited by children with ASD based on the stronger association between teacher ratings on the SRS and the ADOS compared to that between the teacher SRS and the ADI-R. Szatmari et al. [45] warned that this discrepancy between home and school might lead to higher parental stress. Thus, we should exercise caution when interpreting information from parents and teachers in diagnosis and assessment.

Regarding gender differences, it appears that teachers tend to rate girls with ASD lower than boys with ASD, whereas they rate girls without ASD higher than boys without ASD, although these differences reached statistical significance only on the social awareness subscale of the teacher report. Similar gender differences were reported in Norway for total population data using the ASSQ [19]. By contrast, in a Mexican sample [31], affected girls scored higher than affected boys on the teacher-report SRS, whereas control boys scored higher than control girls. However, closer inspection revealed a similar gender difference related to the social awareness subscale between ours and Fombonne et al. [31]. In both studies, teacher and parent ratings for girls did not agree on this subscale, and gender differences in teacher ratings were pronounced on this subscale. In this study, teacher ratings on this subscale also did not discriminate girls with ASD from those without ASD. The poor reliability and validity of this subscale might be related to the measurement of social awareness, which is not overt and is difficult to observe from the outside. Lai et al. [46] reported that women with ASD showed fewer autistic features than males but perceived themselves as having more autistic features, perhaps because they are better at hiding their autistic features, or perhaps because of greater self-awareness. Our finding of gender differences, if replicated, emphasizes the need for both a deeper understanding of gender differences in ASD and the establishment of a gender-specific norm.

The ROC analysis demonstrated that teacher ratings on the SRS classified both boys and girls with moderate accuracy, although the parent-report SRS appears to be more accurate than the teacher-report SRS as a screening tool. The optimal cutoff for boys was 58.0 in this study, which fell within the range of 51.5 to 64.5 proposed in previous studies of the teacher-report SRS [17, 31, 34, 44], whereas that for girls was 43.0 in our sample, which fell below the range. If this great discrepancy in cutoff scores between boys and girls is replicated in a different Japanese sample, the importance of establishing gender-specific norms for this population should be emphasized again. In this study, either sensitivity or specificity values were lower compared to those in studies that included only children with ASD and typically developing children [17, 31], which is consistent with studies that included children with non-ASD clinical conditions [34, 44]. Children with non-ASD psychiatric diagnoses such as ADHD or mood disorders tended to have high SRS scores [47, 48], and there is an overlap in SRS scores of children with ASD and those of children with non-ASD psychiatric diagnoses [27]. That is, the sensitivity or specificity values in our sample might be associated with the type of non-ASD controls, including children with non-ASD psychiatric diagnoses whose mean SRS scores were expected to be higher than those of the normative sample.

Regarding cultural differences in teacher ratings of children with ASD, our female sample with ASD scored similar to children with ASD (86.5% male) in a large-sized study by Schanding et al. [17], whereas our male sample with ASD scored higher. However, our sample with ASD of either gender scored lower than children with ASD in other studies [31, 34]. This variance might be partly explained by the sampling method rather than culture-related differences, taking the heterogeneity of ASD into account. As for gender differences found in this study, little evidence exists, except that in a Mexican sample [31], to draw any conclusion about it from a cultural perspective. If our findings on gender differences are replicated in samples representing different cultures, we should consider culture-free gender differences. Or, if our findings are limited to a Japanese sample, we should consider any cultural factor such as social expectations of the female role in public settings, especially in terms of social awareness. Again, cross-cultural validity of the teacher-report SRS would be guaranteed if it is applied to children according to culturally calibrated gender-specific norms.

The major limitation of this study is its small sample size. Further, we did not use the same assessment battery to determine ASD status for children diagnosed with ASD. Comorbid psychiatric disorders were not assessed using diagnostic measures for 23 children with ASD. The strength of this study is that ASD was excluded for all of the non-ASD children.

## 5. Conclusions

In conclusion, this study provided evidence that the teacher-report SRS is a useful measurement of autistic severity with good reliability and validity and recapitulated what has been observed in studies conducted in other countries. Although parent-teacher agreement on the SRS was satisfactory, characteristic discrepancies specific to ASD diagnostic status and gender between informants should be kept in mind when interpreting the SRS from only one-sided informants. To improve sensitivity for children who are at higher risk, especially girls who are likely to remain undiagnosed, we emphasize the importance of combining information from multiple informants and using standardized norms specific to gender, informant, and culture for screening, clinical, or research purposes.

## Figures and Tables

**Table 1:** Mean raw SRS scores of teacher ratings of children with ASD and without ASD (*N* = 130).

Subscale	Boys (*n* = 90)			Girls (*n* = 40)		
	ASD (*n* = 51) M (SD)	Non-ASD (*n* = 39) M (SD)	*t*	ASD (*n* = 19) M (SD)	Non-ASD (*n* = 21) M (SD)	*t*
Awareness	11.6 (0.5)	7.4 (3.6)	4.8^a^	8.7 (3.9)	7.1 (4.3)	1.3^d^
Cognition	14.8 (6.2)	9.3 (6.0)	4.2^a^	12.6 (4.9)	7.3 (5.3)	3.3^b^
Communication	27.3 (10.2)	16.8 (10.0)	4.9^a^	24.7 (12.7)	13.1 (10.7)	3.1^b^
Motivation	11.0 (5.2)	8.4 (5.7)	2.2^c^	10.8 (5.2)	8.4 (6.0)	1.4^d^
Mannerisms	13.9 (8.5)	7.2 (5.4)	4.5^a^	11.3 (8.4)	5.5 (6.4)	2.5^c^

Total	78.6 (29.9)	49.1 (26.9)	4.8^a^	68.2 (28.8)	41.5 (30.2)	2.9^a^

*Note.* SRS: Social Responsiveness Scale; ASD: autism spectrum disorder.

^a^ *P* < 0.001. ^b^ *P* < 0.01. ^c^ *P* < 0.05. ^d^ns.

**Table 2:** Demographic characteristics of 109 children rated by both teacher and parent.

	ASD (*n* = 57)	Non-ASD (*n* = 52)	
		Neuropsychiatric diagnosis (*n* = 19)	TD (*n* = 33)
Boy : girl	41 : 16	12 : 7	20 : 13
Age (years)			
Mean (SD), range	8.60 (3.90), 4–17	8.26 (2.77) 5–15	7.67 (2.13) 5–12
Intellectual level (*n*)			
Normal	34	9	33
Borderline	14	5	0
Mild MR	3	4	0
Moderate MR	2	0	0
Severe MR	2	1	0
MR (unknown level)	2	0	0
IQ*	*n* = 49	*n* = 19	*n* = 32
Mean (SD), range	91.2 (26.8), 31–148	82.7 (23.3), 27–113	109.7 (13.8), 85–146

*Note.* Between the ASD and non-ASD groups, no significant differences existed in gender ratio (*χ* ^2^ = 0.25, ns) or age (*t* = 1.2, ns). The proportion of intellectual level did not differ significantly by group (*χ* ^2^ = 9.4, ns). For 100 children with available IQ data, mean IQ did not significantly differ between groups (91.2 [26.8] for ASD, 99.7 [22.0] for non-ASD). Among the ASD and two non-ASD groups, no significant differences existed in gender ratio (χ^2^ = 0.51, ns) or age (*F *= 0.84, ns). The proportion of intellectual level differed significantly by group (χ^2^ = 28.5, *P* < 0.005). *For 100 children with available IQ data, mean IQ of the ASD group (*n* = 49) and that of the non-ASD neuropsychiatric diagnosis group (*n* = 19) were lower than that of the TD group (*n* = 32) (*t* = 4.1, 4.6, respectively, *P* values < 0.001), whereas no significant difference existed between the former two groups (*t *= 1.2, ns). MR: mental retardation; ASD: autism spectrum disorder; TD: typically developing.

**Table 3:** Intraclass correlation coefficients (*N* = 109).

Teacher rating	Parent rating					
	Awareness	Cognition	Communication	Motivation	Mannerisms	Total
Awareness	Total 0.38^a^					
	Boys 0.50^a ^					
	Girls 0.08^d^					
Cognition		Total 0.45^a^				
		Boys 0.46^a^				
		Girls 0.41^b^				
Communication			Total 0.45^a^			
			Boys 0.45^a^			
			Girls 0.38^c^			
Motivation				Total 0.48^a^		
				Boys 0.47^a^		
				Girls 0.53^a^		
Mannerisms					Total 0.38^a^	
					Boys 0.38^a^	
					Girls 0.29^c^	

Total						Total 0.48^a^
						Boys 0.50^a^
						Girls 0.40^b^

*Note.* This subsample (*N* = 109) comprises 57 children with ASD and 52 children without ASD.

^a^ *P* < 0.001. ^b^ *P* < 0.01. ^c^ *P* < 0.05.^d^ ns.

**Table 4:** Mean raw SRS scores of parent and teacher ratings of children with ASD and without ASD (*N* = 109).

Rater	Boys (*n* = 73)			Girls (*n* = 36)		
	Parent Mean (SD)	Teacher Mean (SD)	*P *	Parent Mean (SD)	Teacher Mean (SD)	*P *
ASD (*n* = 57)	Boys (*n* = 41)			Girls (*n* = 16)		
Awareness	11.9 (3.4)	11.2 (4.3)	ns	10.2 (2.6)	8.6 (4.0)	ns
Cognition	16.2 (6.4)	14.2 (6.0)	ns	16.9 (4.8)	11.6 (4.7)	<0.005
Communication	30.4 (11.3)	26.6 (9.8)	=0.06	30.6 (9.2)	22.4 (12.0)	<0.05
Motivation	12.3 (5.8)	10.6 (5.3)	ns	12.5 (5.4)	10.8 (5.6)	ns
Mannerisms	16.4 (7.8)	12.7 (8.4)	<0.05	14.9 (7.4)	8.9 (6.5)	<0.05

Total	87.2 (31.3)	75.3 (29.2)	<0.05	85.1 (25.3)	62.3 (27.1)	<0.01

Non-ASD (*n* = 52)	Boys (*n* = 32)			Girls (*n* = 20)		
Awareness	6.6 (5.1)	7.4 (3.4)	ns	6.6 (3.3)	7.0 (4.4)	ns
Cognition	8.3 (4.4)	9.5 (6.0)	ns	6.6 (3.9)	7.5 (5.3)	ns
Communication	13.6 (7.0)	17.3 (10.2)	ns	10.8 (6.5)	13.7 (10.6)	ns
Motivation	7.3 (3.7)	8.7 (5.8)	ns	6.3 (4.9)	8.5 (6.1)	ns
Mannerisms	6.2 (4.5)	7.5 (5.5)	ns	4.2 (4.0)	5.8 (6.5)	ns

Total	42.0 (18.7)	50.5 (27.6)	ns	34.3 (19.9)	42.5 (30.6)	ns

*Note.* SRS: Social Responsiveness Scale; ASD: autism spectrum disorder.
